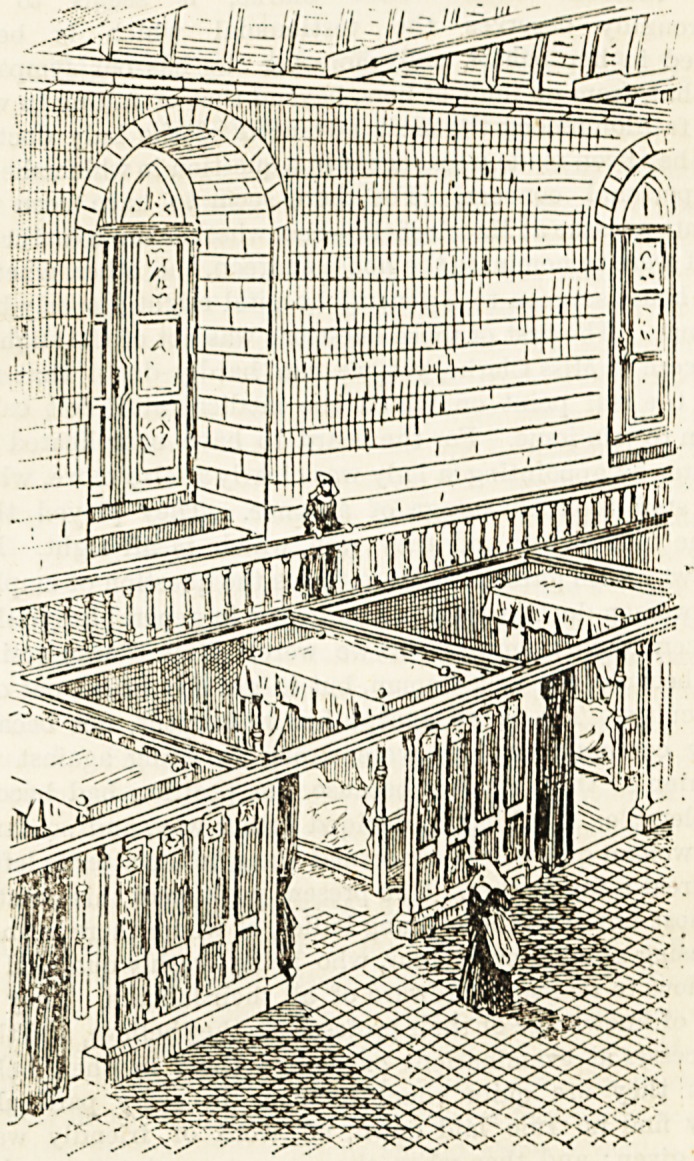# Mediæval Hospitals in France

**Published:** 1902-01-04

**Authors:** 


### MEDIÆVAL HOSPITALS IN FRANCE.

THE hospitals founded in France in the Middle Ages were a hobby of the great lords, to whom they owe their origin, and they surprise one by the enlightened views manifested in their construction. Built on a scale not inferior to that on which the great churches and abbeys of the period were planned, they form a marked contrast to the hospitals of the seventeenth and eighteenth centuries. They were lofty, well lighted, and of massive construction, and the internal fittings were almost luxurious. Unlike the terrible state of things at the Hôtel Dieu in Paris in the eighteenth century, when several patients occupied one bed, the beds were placed in cubicles, with wooden partitions, and were each occupied by one patient only. In that at Tonnerre, a gallery ran round the ward, from which the cubicles could be overlooked by the attendants, and which sheltered the patients from glare and draughts from the tall windows above. These could be opened when necessary, and holes in the window-panes provided constant ventilation. The ward is 90 yards long by 19 yards wide, and is proportionately lofty, with a timbered ceiling. It was provided for forty patients only.

This hospital was built by Marguerite of Burgundy in the thirteenth century. In the sixteenth century hospital at Beaune the beds were placed lengthways in cubicles, and were furnished with handsome canopies and curtains. They stood on wooden platforms, raised 6 inches off the paved floor, and a passage ran between the beds and the wall.

## Figures and Tables

**Figure f1:**